# Cytotoxicity of Different Excipients on RPMI 2650 Human Nasal Epithelial Cells

**DOI:** 10.3390/molecules21050658

**Published:** 2016-05-18

**Authors:** Tamás Horváth, Csilla Bartos, Alexandra Bocsik, Lóránd Kiss, Szilvia Veszelka, Mária A. Deli, Gabriella Újhelyi, Piroska Szabó-Révész, Rita Ambrus

**Affiliations:** 1 Department of Pharmaceutical Technology, University of Szeged, Eötvös u. 6, Szeged H-6720, Hungary; horvath.tamas@pharm.u-szeged.hu (T.H.); bartoscsilla@pharm.u-szeged.hu (C.B.); bocsik@brc.hu (A.B.); kisslori@gmail.com (L.K.); rita-techno@freemail.hu (G.Ú.); revesz@pharm.u-szeged.hu (P.S.-R.); 2 Goodwill Pharma Ltd., Cserzy Mihály u. 32, Szeged H-6724, Hungary; 3 Institute of Biophysics, Biological Research Centre of the Hungarian Academy of Sciences, Temesvári körút 62, Szeged H-6726, Hungary; veszelka.szilvia@brc.mta.hu (S.V.); deli.maria@brc.mta.hu (M.A.D.)

**Keywords:** nasal, cytotoxicity, nasal formulation, MTT dye assay, real-time impedance analysis

## Abstract

The nasal route receives a great deal of attention as a non-invasive method for the systemic administration of drugs. For nasal delivery, specific formulations containing excipients are used. Because of the sensitive respiratory mucosa, not only the active ingredients, but also additives need to be tested in appropriate models for toxicity. The aim of the study was to measure the cytotoxicity of six pharmaceutical excipients, which could help to reach larger residence time, better permeability, and increased solubility dissolution rate. The following excipients were investigated on RPMI 2650 human nasal septum tumor epithelial cells: β-d-mannitol, sodium hyaluronate, α and β-cyclodextrin, polyvinyl alcohol and methylcellulose. 3-(4,5-dimethyltiazol-2-yl)-2,5-diphenyltetrazolium bromide (MTT) dye conversion assay and real-time impedance analysis were used to investigate cytotoxicity. No excipient showed toxicity at 0.3% (*w*/*v*) concentration or below while 1% concentration a significantly reduced metabolic activity was measured by MTT assay for methylcellulose and cyclodextrins. Using impedance measurements, only β-cyclodextrin (1%) was toxic to cells. Mannitol at 1% concentration had a barrier opening effect on epithelial cells, but caused no cellular damage. Based on the results, all additives at 0.3%, sodium hyaluronate and polyvinyl alcohol at 1% concentrations can be safely used for nasal formulations.

## 1. Introduction

Interest in intranasal administration as a non-invasive route for drug delivery continues to grow rapidly. The high vascularization of the nasal mucosa affords a rapid onset of therapeutic effect and it can be administered easily by the patient or a physician. There is no hepatic first-pass metabolism, and nasal preparations do not require sterile preparations. The easy and non-invasive application maximizes patient comfort and compliance. A large variety of therapeutic compounds can be delivered intranasally, including relatively large biomolecules such as peptides and proteins, particularly in the presence of permeation enhancers [1]. Drugs can cross through the nasal mucosal membrane using two different pathways: transcellularly—across the cell—and paracellular, via the intercellular tight junctions. Nasal delivery requires special formulations and vehicles [2,3] to achieve the right bioavability. Excipients, such as absorption enhancers or mucoadhesive polymers, can increase the drug residence time in the nasal cavity and enhance the absorption [4,5]. Because of the sensitiveness of the respiratory mucosa, not only the active ingredients but the additives are needed to be tested in adequate models for real toxicity data [6]. Our groups have recently optimized a human nasal epithelial cell based culture model and tested the cellular toxicity of sucrose esters, novel pharmaceutical additives [7,8].

The aim of our present work was to investigate the effect of the most frequently used excipients on RPMI 2650 human nasal epithelial cells by two different methods. The colorimetric endpoint MTT dye conversion assay provided information on the metabolic state of the cells, while real-time monitoring of impedance showed the biological state of the cell cultures including growth, viability and adherence without using a tracer. Previous measurements related to this topic cannot be found in the literature.

## 2. Results and Discussion

**Figure 1:**
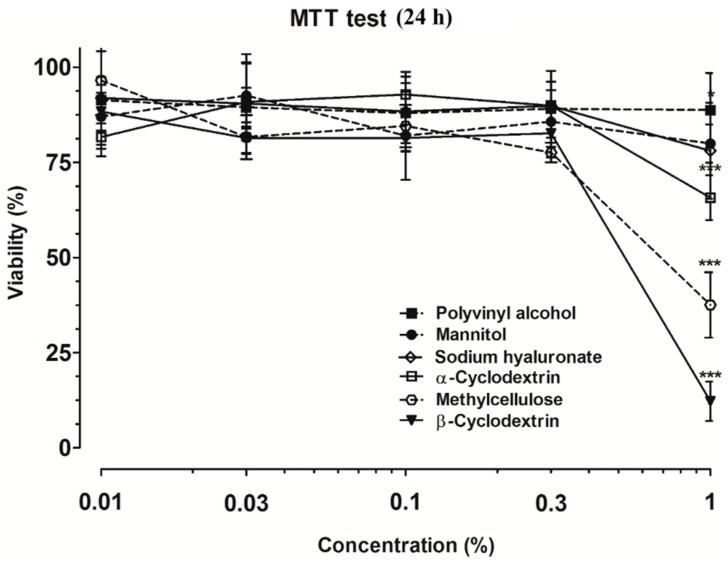
Toxicity of different excipients on RPMI 2650 cells measured by 3-(4,5-dimethyltiazol-2-yl)-2,5-diphenyltetrazolium bromide (MTT) dye conversion assay. All values presented are means ± SD, *n* = 4. Significant differences compared to the values measured in control group are indicated as * *p* < 0.05; *** *p* < 0.001.

Based on the MTT assay, none of the excipients showed toxicity at 0.3% (*w*/*v*) concentration or below on human nasal epithelial cells. At 1% concentration, a highly and significantly reduced metabolic activity was measured by the colorimetric test of methylcellulose and the cyclodextrins (Figure 1). This concentration of the excipients was further studied in a kinetic experiment using impedance measurements.

**Figure 2:**
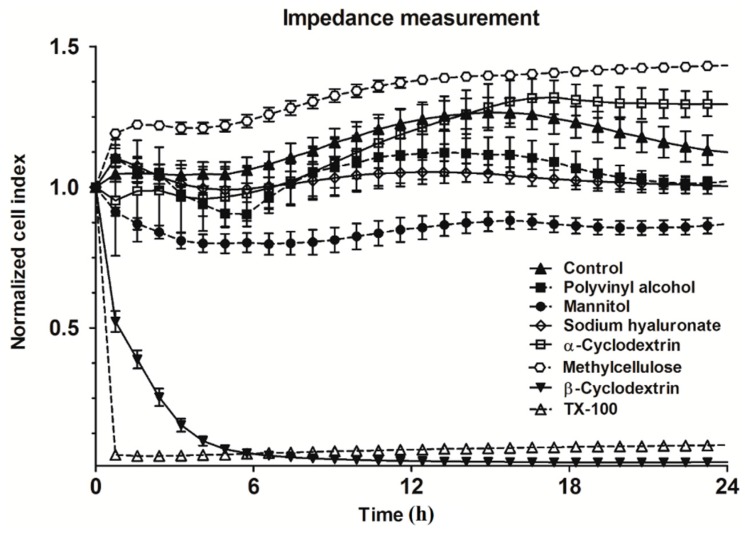
Changes in cell index indicating viability of RPMI 2650 cells treated with different excipients for 24 h and measured by real-time cell impedance analysis. All values presented are means ± SD, *n* = 8. TX-100: 1% Triton X-100.

The results of the real-time cell-analysis with excipients used at 1% concentration are shown in Figure 2.

Triton X-100 immediately and irreversibly dropped the impedance of the cell layers, indicating cell death. Using this method, β- was toxic but α-cyclodextrin was not toxic on RPMI 2650 cells. Mannitol decreased the impedance of the layers, indicating opening of intercellular junctions. The effect of hyperosmotic mannitol on intercellular junctions has been already described for different barriers, including the blood-brain barrier [4]. The other additives showed no cytotoxicity by the kinetic measurement. Polyvinyl alcohol at 1% was not toxic in any of the tests, indicating its safety, which was already confirmed in animal studies [9]. A similar result was obtained for sodium hyaluronate. Our previous study also highlighted the safe use of nasal application of hyaluronate as mucoadhesive excipient in rats [5]. Based on the MTT assay, mannitol at 1% concentration had no effect on the viability of RPMI 2650 cells, but the decreased impedance implied some effects on the intercellular junctions [4]. In the case of the methylcellulose, the cell metabolic assay did not show cell damages, but the impedance measurement did. The observed high viscosity of 1% methylcellulose may affect the endocytosis of MTT dye, the exocytosis of formazan crystals and maybe influenced the colorimetric assay. In case of both assays β-cyclodextrin showed the most toxic among the tested additives. This result was in agreement with the toxicity of cyclodextrins on Calu-3 epithelial cells [10].

## 3. Experimental Section

### 3.1. Materials

Six different auxiliary agents, β-d-mannitol, an osmotic additive, sodium hyaluronate, a mucoadhesive polymer, α- and β-cyclodextrins, absorption enhancers, methylcellulose and polyvinyl alcohol, viscosity-increasing materials were tested at five different concentrations between 0.01% and 1% (*w*/*v*), on RPMI 2650 cells. Regarding the literature, the chosen excipients were used in different nasal forms earlier. Table 1 summarizes the excipients from the nasal forms, concentration of the auxiliary agents and their role in the compositions.

All reagents were purchased from Sigma-Aldrich Ltd. (Budapest, Hungary), unless otherwise indicated. β-d-mannitol was from Hungaropharma Plc. (Budapest, Hungary), sodium hyaluronate from Gedeon Richter Plc. (Budapest, Hungary), α- and β-cyclodextrins from Cyclolab Ltd. (Budapest, Hungary), polyvinyl alcohol 27,000 from ISP Customer Service GmBH (Cologne, Germany) and methylcellulose purchased from HARKE Pharma (Muelheim an der Ruhr, Germany). Stock solutions were prepared by dissolving compounds Eagle’s minimal essential medium.

### 3.2. Methods

#### 3.2.1. MTT Dye Assay 

RPMI 2650 (ATCC cat. no. CCL 30) cells were grown in Eagle’s MEM supplemented with 10% foetal bovine serum and 50 μg/mL gentamicin in a humidified 37 °C incubator with 5% CO_2_ [6,7]. The cells were cultured in 96-well plates coated with 0.05 % rat tail collagen. After treatments with the excipients the cells were incubated with 0.5 mg/mL MTT solution for 3 h in CO_2_ incubator. The amount of formazan crystals was dissolved in dimethyl-sulfoxide and determined by measuring of absorbance at 570 nm with a microplate reader (Fluostar Optima, BMG Labtechnologies, Ortenberg, Germany). The cell viability was expressed as the percentage of the MTT reduction by RPMI 2650 cells treated with culture medium (negative control, 100 % viability).

#### 3.2.2. Real-Time Impedance Measurement 

Living cells were monitored by real-time cell electronic sensing (RTCA-SP, ACEA Biosciences, San Diego, CA, USA) The device utilizes an electronic readout called impedance to non-invasively quantify adherent cell proliferation and viability as used in our previous studies [7,8]. RPMI 2650 cells were seeded in 96-well plates containing gold microelectronic sensor arrays coated with rat-tail collagen (0.05%) and grown at 37 °C in an incubator. Before measurements, cell-free medium was used to determine the background impedance. Twenty-four hours later, the cells were treated with additives. Impedance was measured before the treatment in every 5, and during the treatment in every 2 min. The normalized cell index is determined by the formula:

(R_n_ − R_b_)/15 (1)

where R_n_ is the impedance between cells and electrode, R_b_ is background impedance of the well with the media alone. 

#### 3.2.3. Statistical Analyses 

All data presented are means ± SD. Experiments were performed at least 3 times with 4–8 parallel samples. Values were compared using the analysis of variance followed by Dunnett tests (GraphPad Prism 5.0 software, WaveMetrics Inc., Portland, OR, USA). Changes were considered statistically significant at *p* < 0.05.

## 4. Conclusions

Because earlier published research works did not contain cytotoxicity tests, our aim was to determined applicable concentrations of the frequently applied experiments in nasal formulation. In conclusion of the results of MTT and impedance measurements, polyvinyl alcohol and sodium hyaluronate at 1% concentrations can be safely used for nasal formulations. β-d-mannitol, α- and β-cyclodextrins and methylcellulose are suggested to be used in 0.3% concentrations in nasal vehicles. Choosing the excipients within the above mentioned concentrations appears promising for minimizing toxicity. Also, there is a possibility to use them for local or systemic nasal drug development.

## Figures and Tables

**Table 1:** Important additives in nasal formulations.

Excipient	Role in Nasal Form	Concentration	Ref.
β-d-mannitol	osmotic additive	12% *w*/*v*	[11]
sodium hyaluronate	absorption enhancer	0.1%–1.5% *w*/*v*	[12]
polyvinyl alcohol	viscosity-increasing material	4% *w*/*v*	[13]
α-cyclodextrins	absorption enhancer	5% *w*/*v*	[14]
β-cyclodextrins	absorption enhancer	5%–30% *w*/*v*	[15]
methylcellulose	viscosity enhancing agent	0.1%–1% *w*/*v*	[16]
